# Herpes virus seroepidemiology in the adult Swedish population

**DOI:** 10.1186/s12979-017-0093-4

**Published:** 2017-05-10

**Authors:** Jan Olsson, Eloise Kok, Rolf Adolfsson, Hugo Lövheim, Fredrik Elgh

**Affiliations:** 1 Department of Clinical Microbiology, Virology, Umeå University, Umeå, Sweden; 2 Department of Forensic Medicine, University of Tampere, Tampere, 33520 Finland; 3 Department of Clinical Sciences, Psychiatry, Umeå University, Umeå, Sweden; 4 Department of Community Medicine and Rehabilitation, Geriatric Medicine, Umeå University, Umeå, Sweden

**Keywords:** Herpes, Herpes simplex, Cytomegalovirus, Varicella zoster virus, Seroprevalence, Epidemiology

## Abstract

**Background:**

Herpes viruses establish a life-long latency and can cause symptoms during both first-time infection and later reactivation. The aim of the present study was to describe the seroepidemiology of Herpes simplex type 1 (HSV1), Herpes simplex type 2 (HSV2), Cytomegalovirus (CMV), Varicella Zoster virus (VZV) and Human herpes virus type 6 (HHV6) in an adult Swedish population (35–95 years of age).

**Methods:**

Presence of antibodies against the respective viruses in serum from individuals in the Betula study was determined with an enzyme-linked immunosorbent assay (ELISA). Singular samples from 535 persons (53.9% women, mean age at inclusion 62.7 ± 14.4 years) collected 2003-2005 were analyzed for the five HHVs mentioned above. In addition, samples including follow-up samples collected 1988–2010 from 3,444 persons were analyzed for HSV.

**Results:**

Prevalence of HSV1 was 79.4%, HSV2 12.9%, CMV 83.2%, VZV 97.9%, and HHV6 97.5%. Herpes virus infections were more common among women (*p* = 0.010) and a lower age-adjusted HSV seroprevalence was found in later birth cohorts (*p* < 0.001). The yearly incidence of HSV infection was estimated at 14.0/1000.

**Conclusion:**

Women are more often seropositive for HHV, especially HSV2. Age-adjusted seroprevalence for HSV was lower in later birth cohorts indicating a decreasing childhood and adolescent risk of infection.

## Introduction

Human herpesviruses (HHV1-8) are ubiquitous human pathogens with a global distribution. Epidemiological studies have identified geographic location, socioeconomic status, and age as primary factors for acquisition of HHV infection [1]. The infection cycle involves a primary infection, followed by a latency phase that may be interrupted by episodes of reactivated infection [1]. Although this pattern of infection is shared by all HHVs, variation among the included viral species exists e.g. concerning the tissue involved in the latency phase of the infection. HHV1-3 (HSV1, HSV2 and VZV) establish latency in sensory ganglia, while the other HHVs (EBV, CMV, HHV6-8) employ lymphocytes, monocytes, and sometimes also epithelium for latency. When reactivated, HHV1-3 spread along nerves, HHV4 (EBV), HHV7 and HHV8 expand in lymphocyte populations, while HHV5 (CMV) and HHV6 often spread systemically [1]. HHVs have been attributed a role in the development of chronic disorders, such as Alzheimer’s disease [2–6], cardiovascular disease [7–9], cognitive impairment [10, 11], and depression [12–14]. We, and others researching the field of late neurological sequela from HHV infections have had great use of serological screening in cohorts of adult and elderly individuals [5, 6, 15–18]. Although literature on the seroepidemiology of HHV infections is extensive, most studies focus on young individuals or selected populations posing already known risks from HHV infections [19–22]. We therefore performed the present study with the aim to estimate the prevalence of HHV1 (HSV1), HHV2 (HSV2), HHV3 (VZV), HHV5 (CMV), and HHV6 in serum samples retrieved from individuals in the Betula study [23], reflecting a population of adults, including the elderly, in Sweden. For HSV1&2 combined, we further analyzed a larger cohort with longitudinal samples allowing for estimation on the trend in yearly incidence.

## Methods

### Participants

The Betula study is an ongoing longitudinal, prospective cohort study with the overall aim of investigating how memory function and health develop across the adult life span [23]. The study is designed as a mixed cohort and cross-sectional study, modeled after Schaie [24, 25], to enable the separation of age, cohort and time of measurement effects.

The Betula study started in 1988 by recruiting 1,000 persons from the municipality of Umeå – a municipality of about 120,000 inhabitants located in Northern Sweden. The participants were randomly selected from the Swedish Population Registry, and were invited to participate via an introductory letter and a follow-up telephone call. Recruitment continued until participants within all age groups were fully enrolled. In the first wave, 1,976 persons were contacted to obtain 1,000 participants. To fulfill the primary study aims, persons with severe visual or auditory deficits, cognitive deficits due to intellectual disability, severe psychiatric illness, suspected dementia, and those who did not speak and understand the Swedish language were excluded.

The first cohort (sample 1; S1) of 1,000 persons was investigated in 1988 – 1990 (time-point 1; T1), and was followed-up every five years thereafter until 2008 – 2010 (T2 to T5). Additional cohorts, from the same geographical region were included at each subsequent wave of investigation (T2 to T5). At T2 (1993 – 1995) two cohorts (S2, *n* = 997, and S3, *n* = 966) were enrolled, at T3 (1998 – 2000) one cohort of 563 persons (S4), and T4 (2003 – 2005) another cohort of 562 persons (S5) was enrolled.

S1 and S2 comprised persons aged 35, 40, 45, 50, 55, 60, 65, 70, 75 and 80 years at inclusion, with up to 100 individuals in each age group. The S3 cohort comprised persons aged 40 to 85 at inclusion, up to 100 in each age group, and the S4 and S5 cohorts comprised people in 12 different age groups from 35 to 95 years old at inclusion, with up to 50 in each group. The proportion of men and women in each cohort and age group was equal, roughly corresponding to the gender distribution in the general population.

The S3 cohort, like S1, was followed with repeated examinations every five years until T5. A part of the S2 cohort was re-examined at T3 but not thereafter, whereas S4 (1998 – 2000) and S5 (2004 – 2006) were examined only at the time of inclusion.

Samples included for the cross-sectional analysis of anti-HSV1, anti-HSV2, anti-VZV, anti-CMV and anti-HHV6 were all stored serum samples available from S5T4 (*n* = 535, age 35-95, sampling timepoint 2003-2005). One sub-cohort, sampled once, was selected from the Betula study to make assessment of the five HHVs sero-prevalence affordable, and this particular cohort (S5T4) was found suitable because its samples are relatively recent, and it contains participants of a wide age-distribution. Samples included in the additional cross-sectional and longitudinal analysis of anti-HSV were all stored serum samples available from S1-5 T1-5. The total number of participants was 3,444, from which 2,213 contributed one sample, and 1,231 provided two or more samples from different sampling timepoints (1988 – 1990, 1993 – 1995, 1998 – 2000, 2003 – 2005, and 2008 – 2010).

### Serum analyses

Frozen serum samples were thawed and analyzed for anti-HSV, anti-VZV, anti-CMV, and anti-HHV6 IgG antibodies using Enzyme-linked immunosorbent assays (ELISA). In a procedure to separate anti-HSV positive samples into anti-HSV1, anti-HSV2, or anti-HSV1 + 2 positive, anti- HSV positive samples were further analyzed for presence of anti-HSV2 IgG, afterwhich anti-HSV2 positive samples were analyzed for presence of anti-HSV1 IgG. For anti-HSV, anti-VZV, and anti-CMV, ELISA assays developed in-house were used [26–28], for anti-HSV1 and anti-HSV2 HerpeSelect®-assays (Focus diagnostics) were utilised, and for anti-HHV6 the HHV-6 IgG Antibody ELISA Kit (Advanced biotechnologies inc.) was used. For the in-house methods, antigens against HSV, VZV, and CMV were acquired by growth of HSV1 Umeå clinical isolate 3458-13 on GMK cells, VZV strain SMI 1197 on VeroE6 cells, and CMV strain Ad169 on HumB cells, respectively. Plasma incubation on antigen-coated ELISA plates was performed at 4 °C overnight. Analyses were performed in duplicate using uninfected cell extract as a negative control. In each ELISA run, high and low positive controls and a negative control were included to monitor the quality of individual runs and inter-assay variation. Plasma were diluted 1/420 in phosphate buffered saline supplemented with 0.05% (v/v) Tween-20 and 1% dried milk. IgG antibodies were identified using goat F(ab)2 anti-human IgG, alkaline phosphatase conjugate (Invitrogen) diluted 1/6000, and developed using p-nitrophenyl phosphate disodium substrate (Sigma-Aldrich). The IgG antibody activity of the individual sample was expressed in arbitrary units (AU) as a percentage of the net-absorbance at 405 nm (absorbance of virus-coated well minus absorbance of control antigen well) of the positive control. Samples with IgG values of 5 AU or above were regarded as positive for HHV IgG antibody content.

All serological methods were run according to routine analyses performed in the clinical diagnostics lab, which is a part of Norrlands Universitetssjukhus (NUS). The methods are accredited by Swedac according to ISO 17025 standards.

### Statistics

Chi-2 test, independent sample t-test, and Pearson correlation were used for univariate analyses as appropriate. A multiple logistic regression model was used to differentiate between the effect of the variables age, sex, and birth year of HSV IgG seroprevalence. To plot HSV seroprevalence in relation to age, a linear regression model was used to fit regression lines.

The HSV incidence was calculated by dividing the number of new cases with the total follow-up time (person-years) among HSV negative participants. New cases were calculated as the number of seroconvertants subtracted by the number of serorevertants.

*P* < 0.05 was regarded as statistically significant. The SPSS 20.0 software for Mac was used for statistical calculations.

## Results

The seroprevalence of IgG antibodies towards five common human herpes viruses was cross-sectionally investigated in a representative sample (T5 S4) from an adult Swedish population. The sample included 535 people (274 women and 261 men) aged 35 to 95 years (mean age 60.7 ± 16.2 years). The seroprevalence of IgG antibodies against HSV1, HSV2, VZV, CMV and HHV6 are presented in Table 1.

**Table 1:** Herpes virus seroprevalence, *N* = 535

Virus IgG antibodies	N_positive_/N_total_	%	95% confidence interval
Herpes simplex type 1	425/535	79.4	76.0 – 82.9
Herpes simplex type 2	69^a^/535	12.9	10.1 – 15.8
Varicella zoster virus	524/535	97.9	96.7 – 99.1
Cytomegalovirus	445/535	83.2	80.0 – 86.4
Human herpes virus type 6	517/530^b^	97.5	96.2 – 98.9

Note: ^a^Among the 69 HSV2 positive, 50 were HSV1 positive and 16 were HSV1 negative

^b^Five samples were unavailable for HHV-6 analysis

The relationship between age and sex, and the presence of herpes virus antibodies was investigated (Table 2). Women were seropositive for on average 3.8 ± 0.7/5 of the analyzed antibodies, compared to 3.6 ± 0.7/5 for men, *p* = 0.010. Women were more likely to be HSV2- positive compared to men (*p* = 0.013). Age correlated positively with CMV- and HSV1-IgG presence (*p* < 0.001 and *p* < 0.001 respectively), but negatively with HHV6-IgG presence (*p* = 0.034). A relationship between the presence of anti-HSV1 IgG and anti-CMV IgG was found (Pearson’s r 0.167, *p* < 0.001), but not between any other combination.

**Table 2:** Relationship between presence of Herpes virus IgG antibodies, and age and sex

	HSV1	HSV2	VZV	CMV	HHV6
IgG positive_women_, *n* (%)	218 (79.6)	45 (16.4)	269 (98.2)	234 (85.4)	266 (98.9)
IgG positive_men_, *n* (%)	207 (79.3)	24 (9.2)	255 (97.7)	211 (80.8)	251 (96.2)
*p*-value men vs. women	0.943	0.013	0.699	0.159	0.143
Age_IgG positive_, mean ± SD	62.9 ± 16.0	61.5 ± 15.3	60.7 ± 16.1	62.2 ± 16.0	60.3 ± 16.1
Age_IgG negative_, mean ± SD	52.2 ± 14.0	60.6 ± 16.3	61.3 ± 20.1	53.2 ± 15.4	69.9 ± 12.4
*p*-value for difference in mean age	<0.001	0.638	0.902	<0.001	0.034

A larger sample of 3,444 participants from all five cohorts (S1-5 T1-5) (sampled 1988-2010) was investigated for HSV IgG seroprevalence with an ELISA developed in-house. The 535 people in the analyses above were a subsample of the 3,444. All singular samples and the first sample from persons that contributed multiple samples were included. The age ranged between 35 and 95 years, and the mean age was 62.7 ± 14.4. There were 1,860 (54%) women. The anti-HSV IgG seroprevalence was 3,038/3,444 = 88.2%. In this sample HSV was significantly more common among women (1,671 (89.8%) versus 1,367 men (86.3%), p = 0.001), and the mean age of HSV positive individuals was higher, when compared to HSV negative (63.9 ± 14.0 years versus 53.6 ± 14.0 years, *p* < 0.001).

In a multiple linear regression model with age and sex as independent variables, and anti-HSV IgG seropositivity as the outcome variable, the calculated increase in anti-HSV seroprevalence for each subsequent year of age was 0.0051. This value thus corresponds to 0.0051 x 3,444 = 17.6 estimated new cases each year.

**Fig. 1:**
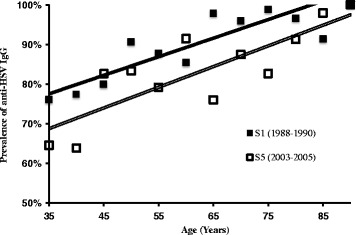
Linear regression model of anti- HSV IgG seroprevalence in relation to age in the S1 (*solid line*) and S5 (*double line*) cohorts. *Squares mark* the mean prevalence in each 5-year age group.

The age, sex, and year of birth of the participants were included in a logistic regression model to individually investigate the effects of these three variables on the outcome variable anti-HSV IgG seropositivity (Table 3). Female sex and earlier year of birth was associated with a higher anti-HSV IgG prevalence, while increasing age was not. The birth cohort effect, giving lower age-specific anti-HSV IgG prevalence in later birth cohorts, is illustrated in Fig. 1 showing HSV IgG seroprevalence in relation to age in two temporally separated study cohorts (S1T1: 1988-1990 and S5T4: 2003-2005).

**Table 3:** Multiple binary logistic regression model of HSV positivity, *N* = 3,444

	Odds ratio	95% confidence interval	*p*-value
Age (years)	1.009	0.989 – 1.030	0.370
Female sex	1.332	1.075 – 1.651	0.009
Year of birth (four digits)	0.958	0.939 – 0.978	<0.001

Samples from 1,231 people who contributed one or several follow-up sample(s) were included in a longitudinal analysis. These people contributed in total 14,089.83 person-years (PY) of follow-up time, defined as the timespan from first sample until the last sample, of which participants who were anti-HSV IgG free at the beginning of each period contributed 1,289.83 PY follow-up time. During the follow-up period, 28 people seroconverted while 10 people seroreverted, hence 28 – 10 = 18 was regarded as the number of incident HSV cases. We treated serorevertants as false negatives and assumed a similar frequency of false-positives. HSV incidence was hence calculated as 18/1,289.83 = 14.0/1000 PY.

In order to discriminate the incidence rate from birth cohort effects, the calculated incidence, 14.0/1000 PY, was compared to the figure of yearly increase for the whole study cohort (17.6).

The 14.0/1000 PY incidence multiplied with 406 anti-HSV IgG negative participants at baseline would predict 5.7 new anti-HSV IgG positive cases during the forthcoming year. New incident cases hence would account for 5.7/17.6 = 32.2% of the observed effect of age to anti-HSV IgG seroprevalence, with the remaining being the birth cohort effect.

## Discussion

We report seroprevalence estimates of five common human herpes viruses in the general adult population in Sweden. The most frequent species, VZV and HHV6, both showed more than 97% prevalence. Studies from USA report similar figures for VZV (99%) [29]. Even HHV6 is reported to be prevalent almost ubiquitously in the adult population [30–33]. We noted a lower prevalence for anti-HHV6 antibodies with increasing age, in line with earlier reports [34, 35]. The prevailing explanation is that the primary infection, and corresponding humoral immunity, almost exclusively occurs in early childhood, and that antibody titers decline with age and thus in some patients goes below our assay’s detection limit [34]. The seroprevalence for CMV was 83%, confirming the high prevalence figures earlier reported from Sweden, regardless of region studied or urbanization status [36–38]. Reports from USA have shown 67%, for a younger cohort [39, 40] and 87% for a cohort of women aged 70––79 [41]. Increasing age is associated with increased seroprevalence for CMV, in line with reports of a significant rate of seroconversion in adults [42]. Seroprevalence for HSV1 was 79%, in good agreement with comparable studies from Switzerland (80%) [43], Sweden (88%) [44] and Finland (86%) [18]. The HSV2 seroprevalence was 13%, placing our cohort in the lower range of comparable earlier studies from Sweden, (16%) [44], or USA, (17%) [45]. The prevalence was significantly higher in women (16%), confirming other cited studies.

Year of birth affected HSV seroprevalence significantly. As illustrated in Fig. 1, the age-specific HSV prevalence is shifted downward in subjects sampled 2003–2005 compared to subjects sampled 1988–1990. When investigated in a logistic regression model, age *per se* had no significant effect on anti-HSV IgG seropositivity. This surprising outcome should be interpreted in the way that in this study cohort - designed to allow separation of the two connected variables age and year of birth - the latter dominates over the former. By analysis of longitudinal samples, the HSV incidence was calculated at 14.0/1000 PY in this population and this incidence rate explains approximately one third of the increase in prevalence by age. The remaining increase can be attributed to year of birth differences in the sub-cohorts, in that later sub-cohorts have a lower prevalence. The year of birth differences could be explained by a decreasing childhood and adolescent risk of HSV, especially HSV1, infection in the population [46, 47]. Changing lifestyle may also influence HSV spread, given its routes of transmission. The lack of analysis on the impact of socio-demographic factors such as level of education and overcrowding, is a limitation of the present study. Further studies and the inclusion of younger participants would be needed to confirm the observation of a decreasing prevalence and could possibly also provide insights on the underlying causes. In light of the accumulating evidence for a role of HSV1 infection in Alzheimer’s disease development [3, 5, 6, 48], it is also worth mentioning that the incidence of dementia is declining in both USA and Sweden [49, 50]. Well-designed comparative studies of age-weighted trajectories will hopefully shed further light on the impact of HHV infectious burden and neuronal damage, leading to sequela such as Alzheimer’s disease.

## Conclusion

Prevalence of HSV1 was 79.4%, HSV2 12.9%, CMV 83.2%, VZV 97.9%, and HHV6 97.5%. Women were more often found to be seropositive for HHV, especially HSV2. Age-adjusted seroprevalence for HSV was lower in later birth cohorts indicating a decreasing childhood and adolescent risk of infection.
